# Prediction of hemodynamic fluctuations after induction of general anesthesia using propofol in non-cardiac surgery: a retrospective cohort study

**DOI:** 10.1186/s12871-018-0633-2

**Published:** 2018-11-10

**Authors:** Sho Kawasaki, Chikako Kiyohara, Shoji Tokunaga, Sumio Hoka

**Affiliations:** 1 Department of Anesthesiology and Critical Care Medicine, Graduate School of Medical Sciences, Kyushu University, Maidashi 3-1-1, Higashi-ku, Fukuoka, Fukuoka Japan; 2 Department of Preventive Medicine, Graduate School of Medical Sciences, Kyushu University, Maidashi 3-1-1, Higashi-ku, Fukuoka, Fukuoka Japan; 3 Medical Information Center, Kyushu University Hospital, Maidashi 3-1-1, Higashi-ku, Fukuoka, Fukuoka Japan

**Keywords:** Propofol, Anesthesia induction, Hemodynamics, Prediction formula

## Abstract

**Background:**

Although propofol is a common anesthetic agent for the induction of general anesthesia, hemodynamic fluctuations are occasionally prominent during induction/intubation. The aims of this study were to determine the influential factors on enhanced hemodynamic fluctuation and to establish a prediction formula to quickly determine the dose of propofol to protect against hemodynamic fluctuations.

**Methods:**

This retrospective cohort study patients (*n* = 2097) were 18 years or older. They underwent general anesthesia induction using propofol and orotracheal intubation for non-cardiac surgery at Kyushu University Hospital during April 2015 to March 2016. Preoperative patient clinical information was collected from anesthesia preoperative evaluation records. Intraoperative data were obtained from computerized anesthesia records. If patients’ post-induction mean arterial blood pressure (MAP) decreased or increased 30% or more from their pre-induction MAP, they were determined to have enhanced hemodynamic fluctuations. Unconditional logistic regression was used to assess the adjusted odds ratios (ORs) and 95% confidence intervals (CIs). Structural equation modeling (SEM) was conducted to simultaneously examine the direct and indirect effect (path coefficient = r) of potential variables.

**Results:**

In the SEM analysis, age was significantly associated with enhanced hemodynamic fluctuations (adjusted odds ratio = 1.008, 95% CI = 1.001–1.015, *P* = 0.03). Age (path coefficient (r) = − 0.0113, 95% CI = − 0.0126–0.010, *P* < 0.001), American Society of Anesthesiologists physical status (ASA-PS) (*r* = − 0.0788, 95% CI = − 0.1431–0.0145, *P* = 0.02), sex (*r* = 0.057, 95% CI = 0.0149–0.9906, *P* = 0.01), and fentanyl dose (*r* = 0.1087, 95% CI = 0.0707–0.1467, *P* < 0.001) influenced the dose of propofol in induction. The prediction formula of “Propofol dose (mg) = [2.374 – 0.0113 × age (year) – 0.0788 (if ASA-PS 3 or 4) + 0.057 (if female) + 0.1087 × fentanyl dose (μg/kg)] × body weight (kg)” was derived.

**Conclusions:**

Age was associated with hemodynamic fluctuations in induction. Although the prediction formula is considered to be acceptable, future studies validating whether it can decrease patients’ risk of enhanced hemodynamic fluctuations in clinical situations are necessary.

## Background

Propofol is a common anesthetic agent for the induction of general anesthesia. Rapid infusion of propofol often causes hypotension [1–5]. Intraoperative hypotension may lead to negative outcomes such as myocardial injury, stroke, acute kidney injury, and death [6–10]. Conversely, laryngoscopy and tracheal intubation often cause hypertension [11–15]. Hypertension associated with intubation may cause negative outcomes such as myocardial infarction, heart failure, pulmonary edema, and cerebral and subarachnoid hemorrhage [16–18]. Thus, preventing hemodynamic fluctuations, or stabilizing hemodynamics, would avoid some harmful complications. The recommended dose of propofol in induction of general anesthesia is 2–2.5 mg/kg.

The American Society of Anesthesiologists physical status (ASA-PS) scale is a widely used grading system to measure the preoperative health of surgical patients. The ASA-PS scale is a subjective assessment of a patient’s overall health that is based on 6 categories (1 [healthy and no complications] to 6 [brain death]). Elderly patients and/or patients with ASA-PS scores of 3 or higher (having moderate to severe comorbidities) require a reduction in dose of propofol due to adverse prognosis [19–21]. However, the relationship between characteristics of patients and anesthesia induction dose of propofol has not yet been fully evaluated.

Since reliable guidelines for determining the propofol dose in anesthesia induction do not exist, this determination has been entrusted to each anesthesiologist. Although anesthetic devices and drugs have undergone improvements, intraoperative hemodynamic fluctuations continue to occur [22]. Structural equation modeling (SEM) is a multivariate statistical tool for evaluating complex relations in several research fields. SEM is a useful method for evaluating simultaneous causal associations among factors, which allows factors to be both dependent and independent. By explicitly accounting for the underlying roles of the factors of enhanced hemodynamic fluctuations, SEM can provide more insight and a better understanding of how risk factors influence the outcome.

The aims of this study were to identify the predictors of hemodynamic fluctuations after induction of general anesthesia and to develop a prediction formula, using SEM analysis, to calculate the appropriate propofol dose in induction.

## Materials and methods

### Patient population and ethics

Our retrospective cohort study protocol was approved by the Institutional Review Board (IRB) of Kyushu University Hospital in March 2018 (IRB# 29–630). The IRB waived the need for written informed consent from participants. Because all patients received usual care and were not subjected to research activities, written informed consent was not obtained. This article adheres to the applicable Strengthening the Reporting of Observational Studies in Epidemiology (STROBE) guidelines.

Patients who were 18 years old or older (range: 18 to 96 years old) and underwent general anesthesia induction using propofol subsequently with tracheal intubation for non-cardiac surgery were included in this study. All patients (*n* = 2760) underwent inpatient anesthesia and surgery at the Kyushu University Hospital in Fukuoka, Japan, during the period April 2015 to March 2016. Patients who were missing important covariates regarding intubation or hemodynamic data were excluded (*n* = 56). Patients who did not receive oral intubation or who underwent repeated attempts at intubation were also excluded (*n* = 607). A total of 2097 participants were included in this analysis.

### Validation study of the prediction formula

In another part of the study, the prediction formula was then applied to 1974 patients, who were admitted to the same hospital during the period April 2016 to March 2017 in order to validate the derived prediction formula. A sample size of 1974 patients was expected to yield sufficient subjects to validate the prediction formula. This separate cohort was comparable in confounders to the study cohort.

### Clinical data

Preoperative patient clinical information was collected from anesthesia preoperative evaluation records (NIHON KOHDEN Corp, Tokyo, Japan). The records consisted of demographic data, laboratory data, and medical history. Intraoperative data were obtained from computerized anesthesia records (NIHON KOHDEN Corp.). These data from the patient monitoring system and anesthesia machine were automatically stored in anesthesia records, whereas drug dose and anesthesia techniques were registered manually. No patients received anesthetic premedication. In the operation room, the patients were placed on the operating table in the supine position. Baseline data, such as electrocardiography, non-invasive blood pressure, and pulse oximetry, were obtained from the records of standard monitors. An invasive arterial catheter was inserted after induction of general anesthesia if necessary. Heart rate was continuously recorded, and non-invasive arterial blood pressure was recorded every 2.5 min. Basically, arterial blood pressure was recorded continuously after insertion of the invasive arterial catheter. Induction of general anesthesia was elicited by propofol, fentanyl, remifentanil, rocuronium, and inhalation anesthetics (sevoflurane or desflurane in oxygen). Propofol was administered according to the instruction manual. Selection of induction drugs and doses depended on the anesthesiologist’s clinical judgment based on the patients’ medical conditions. Laryngoscopy and orotracheal intubation were performed by an attending anesthesiologist (residents or specialists).

### Definition of main outcome

Baseline arterial blood pressure (pre-induction) was defined as the first measurement in the operating room under awake conditions. While, arterial blood pressure after induction (post-induction) was measured within 3 min after tracheal intubation. In case of insertion of an invasive arterial catheter, arterial blood pressure 1 min after tracheal intubation was measured. Several definitions of hemodynamic fluctuations have been previously published. However, Bijker et al. reviewed the major journals and concluded that hypotension is best defined as 20–30% reduction of mean arterial blood pressure (MAP) [22]. In addition, Goldman et al. reported that postoperative cardiac death was significantly associated with a 33% or greater fall in intraoperative systolic blood pressure (SBP) from baseline for more than 10 min [23]. Moreover, Bijker et al. reported that a decrease in MAP of more than 30% from baseline was associated with the occurrence of a postoperative stroke (odds ratio [OR] = 1.013 for every minute of hypotension, 99% confidence interval [CI] = 1.000–1.025) [7]. Based on the results mentioned above, if a patient’s post-induction MAP decreased or increased 30% or more from their pre-induction MAP, they were determined to have enhanced hemodynamic fluctuations before and after anesthesia induction.

### Statistical analysis

Differences in patient characteristics between the fluctuation status categories were compared using the Student t-test for normally distributed continuous variables and the Mann-Whitney U test for not-normally distributed continuous variables. Categorical variables were compared using the Pearson chi-squared test or the Fisher exact test where appropriate. Variables with a *P* value of less than 0.20 in univariate analyses or clinical relevance were entered into the multivariate logistic regression model. Multivariate logistic regression analysis was used to adjust for potential confounding variables and to identify the association of independent variables with hemodynamic fluctuations. Similarly, variables with a *P* value of less than 0.20 in multivariate analysis or clinical relevance were entered into SEM. SEM could be performed to simultaneously examine the direct and indirect effect of potential variables. A path coefficient indicates the direct effect of a variable assumed to be a cause on another variable assumed to be an effect [24]. As path coefficients are estimated from correlations, they should be standardized. In our study, SEM was used to reveal the propofol-mediated effect of potential variables to hemodynamic fluctuations and the effect of potential variables to propofol dose in anesthesia induction. The potential variables are often highly intercorrelated (multicollinearity). Multicollinearity was assessed using variance inflation factor (VIF) [25], which measures the inflation in the variances of the parameter estimates due to multicollinearity potentially caused by correlated predictors. Although there is no consensus as what cutoff based on values of VIF should be used to detect multicollinearity, VIF greater than 5 [25] is suggested for detecting multicollinearity.

To assess the accuracy of the prediction formula, we applied it to a separate cohort of 1974 patients. We defined differences less than 5 mg between the actual dose and estimated dose as “fitted to the formula (fitted group)” and otherwise defined as “unfitted to the formula (unfitted group)”.

Age, height, weight, blood pressure, and drug use except vasopressor at induction and intubation were treated as continuous variables. Remaining covariates, namely ASA-PS scale (scores of 1 or 2 and 3 or 4), obesity (body mass index < 25 kg/m^2^ and ≥ 25 kg/m^2^), anesthesia start time (forenoon and afternoon), a clinical history of comorbidities (positive and negative), vasopressor (use [phenylephrine 0.05–0.1 mg or ephedrine 4-8 mg] or non-use) were treated as categorical variables. The 48 potential predictors as reported in the pre-existing literature, which may be associated with hemodynamic fluctuations in induction of general anesthesia, are shown in Table 1. All *P* values were 2-sided, and those less than 0.05 were considered statistically significant. All statistical analyses were performed using Stata^Ⓡ^ version 14.1 software (StataCorp LP, College Station, Texas, USA).

**Table 1:** Demographic, lifestyle-related, physical and clinical characteristics of perioperative patients according to hemodynamic fluctuation status (*n* = 2097)

Variables	Hemodynamic fluctuation				*P* value
	≥30% (*n* = 482)	No.^a^	< 30% (*n* = 1615)	No.^a^	
Demographic and lifestyle-related					
Age (years), mean (range)	61.2 (18–96)	0	58.3 (18–93)	0	< 0.001
Female sex, *n* (%)	247 (51.2)	0	813 (50.3)	0	0.73
Height (cm), mean (95% CI)	160.5 (159.7–161.4)	0	161.0 (160.6–161.5)	3	0.29
Weight (kg), mean (95% CI)	59.3 (58.1–60.5)	0	60.1 (59.5–60.8)	0	0.23
BMI, *n* (%)		0		3	0.23
< 18	48 (29.6)		114 (70.4)		
≥ 18, < 25	316 (22.4)		1093 (77.6)		
≥25, < 35	110 (22.5)		379 (77.5)		
≥ 35	8 (23.5)		26 (76.5)		
Smoking history, *n* (%)	226 (46.9)	0	721 (44.8)	5	0.42
Physical status					
ASA-PS scale, *n* (%)		0		0	0.52
1 or 2	417 (86.5)		1415 (87.6)		
3 or 4	65 (13.5)		200 (12.4)		
Low exercise tolerability, *n* (%)	32 (6.7)	1	91 (5.7)	5	0.41
Clinical and laboratory data					
Arterial BP (mmHg) in the ward, mea*n* (95% CI)					
Systolic BP	125 (123–126)	15	125 (124–126)	49	0.92
Diastolic BP	74 (73–75)	16	74 (74–75)	51	0.82
History of steroid use, *n* (%)	28 (5.8)	1	96 (6.0)	5	0.91
History of opioid use, *n* (%)	3 (0.6)	1	13 (0.8)	5	1.00^b^
Antithrombotic drug use, *n* (%)	66 (13.7)	1	184 (11.4)	5	0.17
Anemia, *n* (%)	172 (35.8)	1	573 (35.5)	2	0.93
Electrolyte imbalance, *n* (%)	4 (0.8)	3	12 (0.8)	6	0.77^b^
Hypoalbuminemia, *n* (%)	70 (14.6)	3	191 (11.9)	6	0.11
Elevated CRP, *n* (%)	133 (27.9)	5	413 (25.8)	14	0.36

Data are presented as *n* (%) for categorical variables, mea*n* (95%CI) or mea*n* (range) for continuous variables

*BMI* Body mass index, *ASA-PS* American Society of Anesthesiologists physical status, *BP* Blood pressure, *CI* Confidence interval, *CRP* C-reactive protein

^a^Number of missing patients

^b^Fisher’s exact test

## Results

**Fig. 1:**
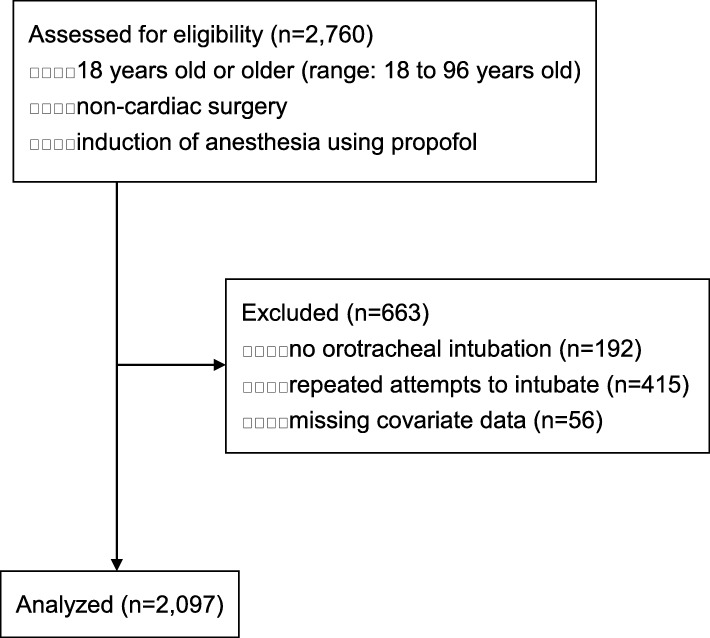
Flow diagram of study subjects. All patients (*n* = 2760) underwent inpatient anesthesia and surgery. All patients were older than 18 years and underwent general anesthesia induction using propofol and tracheal intubation for non-cardiac surgery. Patients who had missing important covariates regarding intubation or hemodynamic data (*n* = 56), who did not receive oral intubation, or who underwent repeated attempts at intubation were excluded (*n* = 607). A total of 2097 participants were included in the analysis

Of 2760 consecutive non-cardiac surgical patients 18 years or older who underwent general anesthesia induction using propofol, 192 did not have orotracheal intubation, 415 underwent repeated attempts at tracheal intubation and 56 were missing important covariate data. Exclusion of these patients resulted in a sample size of 2097 patients (Fig. 1). Enhanced hemodynamic fluctuations after induction of general anesthesia were observed in 482 patients (23.0%). Of these 482 patients, 287 (13.7%) developed hypotension, and 195 (9.3%) developed hypertension.

Tables 1 and 2 show differences in demographics and lifestyle-related and clinical features of the two fluctuation status groups. Age, history of ischemic heart disease, propofol dose, and fentanyl dose were significantly different between the two fluctuation status groups, whereas diabetes mellitus, peripheral vascular disease, and stroke appeared to be marginally significant. However, difference in two clinically important variables, namely ASA-PS score and sex, did not reach statistical significance.

**Table 2:** Clinical history and anesthetic practice of perioperative patients according to hemodynamic fluctuation status (n = 2097)

Variables	Hemodynamic fluctuation				*P* value
	≥30% (*n* = 482)	No. ^a^	< 30% (*n* = 1615)	No. ^a^	
Clinical history, *n* (%)					
Diabetes mellitus	97 (20.1)	0	263 (16.3)	3	0.05
Hyperlipidemia	108 (22.4)	0	309 (19.2)	3	0.12
Hypertension	208 (43.2)	0	673 (41.2)	3	0.58
Ischemic heart disease	33 (6.9)	4	65 (4.0)	7	0.01
Congestive heart failure	5 (1.0)	0	18 (1.1)	3	0.88
Arrhythmia	19 (3.9)	0	70 (4.3)	3	0.70
Valvular heart disease	17 (3.5)	0	76 (4.7)	3	0.27
Cardiomyopathy	1 (0.2)	0	11 (0.7)	3	0.32^b^
Pulmonary hypertension	3 (0.6)	0	7 (0.4)	3	0.71^b^
Peripheral vascular disease	13 (2.7)	0	25 (1.6)	3	0.10
Aortic aneurysm	6 (1.2)	0	9 (0.6)	3	0.12
Stroke	35 (7.3)	0	84 (5.2)	3	0.09
Asthma	32 (6.6)	0	118 (7.3)	3	0.61
Sleep apnea syndrome	22 (4.6)	3	71 (4.4)	12	0.88
Chronic kidney disease	120 (25.1)	3	368 (22.9)	6	0.32
Liver dysfunction	9 (1.9)	5	28 (1.8)	16	0.84
Hyperthyroidism	4 (0.8)	1	12 (0.7)	3	0.85
Hypothyroidism	18 (3.7)	0	47 (2.9)	0	0.36
Rheumatoid arthritis	7 (1.5)	1	16 (1.0)	5	0.39
Gastrointestinal disorder	28 (5.8)	0	92 (5.7)	3	0.93
Operation and anesthesia, *n* (%)					
Emergency surgery	31 (6.4)	0	96 (5.9)	0	0.69
Combined epidural anesthesia	206 (42.7)	0	646 (40.0)	0	0.28
Anesthesia start time, *n* (%)		0		0	0.17
A.M.	294 (61.0)		1041 (64.5)		
P.M.	188 (39.0)		574 (35.5)		
Intubation method, *n* (%)		3		14	0.68
Direct	406 (84.8)		1369 (85.5)		
Video laryngoscope	73 (15.2)		232 (14.5)		
Operator of intubation, *n* (%)		0		0	0.61
Specialist	137 (23.7)		440 (76.3)		
Residents	345 (22.7)		1175 (77.3)		
Drug dosage at induction and intubation, mea*n* (95%CI) or media*n* (range)					
Propofol (mg/kg)	1.8 (1.7–1.8)	0	1.9 (1.9–1.9)	0	< 0.001
Fentanyl (μg/kg)	1.2 (1.2–1.3)	0	1.3 (1.3–1.3)	0	0.02
Remifentanil (μg kg^−1^ min^−1^)	0.2 (0–0.5)	0	0.2 (0–0.5)	0	0.29
Sevoflurane (%)	0 (0–3.3)	0	0 (0–6.2)	0	0.70^c^
Desflurane (%)	0 (0–5.0)	0	0 (0–8.0)	1	0.74^c^
Midazolam (%)	0 (0–5.0)	0	0 (0–4.0)	1	0.17^c^
Vasopressor, *n* (%)	19 (3.9)	0	51 (3.2)	0	0.40

Data are presented as *n* (%) for categorical variables, mea*n* (95%CI) or media*n* (range) for continuous variables

*A.M*. Ante meridian, *P.M.* Post meridian, *CI* Confidence interval

^a^Number of missing patients

^b^Fisher’s exact test

^c^Mann-Whitney U test

Variables with a *P* value of less than 0.20 in univariate analyses or two clinically important variables were entered into the multivariate logistic regression model. The results of multivariate logistic regression analysis are shown in Table 3. Three potentially relevant (*P* < 0.20) variables with hemodynamic fluctuations, namely, ischemic heart disease (adjusted OR = 1.51, 95% CI = 0.93–2.46, *P* = 0.10), propofol dose (adjusted OR = 0.81, 95% CI = 0.65–1.01, *P* = 0.06), and fentanyl dose (adjusted OR = 0.84, 95% CI = 0.69–1.02, *P* = 0.08), were extracted.

**Table 3:** Association between enhanced hemodynamic fluctuation (≥30%) and selected clinical factors

Variables	OR (95% CI)			
	Crude	*P* value	Adjusted^a^	*P* value
Age (year)	1.01 (1.01–1.02)	< 0.001	–	
Female sex	1.04 (0.85–1.27)	0.73	–	
ASA-PS scale, 3 or 4 vs. 1 or 2	1.10 (0.82–1.49)	0.53	0.87 (0.62–1.24)	0.45
Anesthesia start time, P.M. vs. A.M.	1.16 (0.94–1.43)	0.17	1.14 (0.92–1.41)	0.22
Ischemic heart disease	1.76 (1.14–2.71)	0.01	1.51 (0.93–2.46)	0.10
Peripheral vascular disease	1.76 (0.89–3.47)	0.11	1.39 (0.67–2.88)	0.38
Aortic aneurysm	2.25 (0.80–6.34)	0.14	1.55 (0.51–4.70)	0.44
Stroke	1.42 (0.95–2.14)	0.10	1.23 (0.77–1.95)	0.38
Diabetes mellitus	1.29 (0.99–1.67)	0.06	1.12 (0.85–1.48)	0.41
Hyperlipidemia	1.22 (0.95–1.56)	0.12	1.03 (0.79–1.35)	0.82
Hypoalbminemia	1.27 (0.95–1.71)	0.12	1.17 (0.85–1.61)	0.34
Antithrombotic drug use	1.23 (0.91–1.67)	0.18	0.91 (0.63–1.32)	0.62
Propofol (mg/kg)	0.71 (0.59–0.87)	< 0.001	0.81 (0.65–1.01)	0.06
Fentanyl (μg/kg)	0.80 (0.66–0.96)	0.02	0.84 (0.69–1.02)	0.08
Midazolam (mg)	0.75 (0.47–1.20)	0.18	0.79 (0.49–1.27)	0.33

^a^Mutually adjusted for all variables in Table 3

*ASA-PS* American Society of Anesthesiologists physical status, *OR* Odds ratio, *CI* Confidence interval

**Fig. 2:**
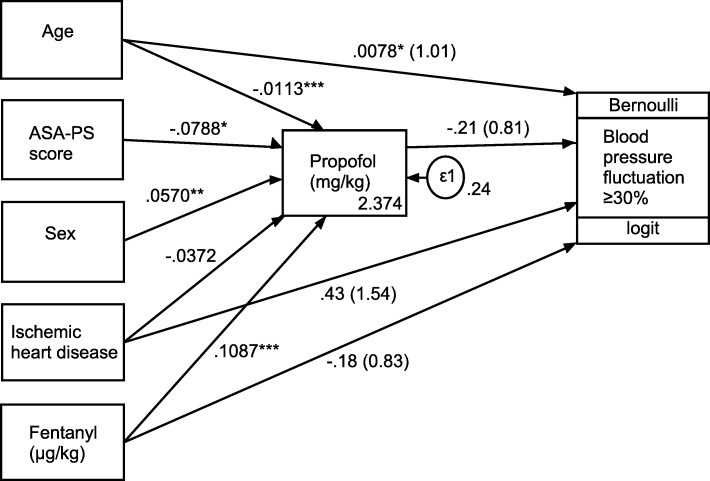
Factors predicting enhanced blood pressure fluctuation with path coefficients and odds ratios (in parentheses) (*n* = 2097). ASA-PS American Society of Anesthesiologists physical status. Numbers by the arrow lines represent the estimated coefficients with significance level: *P < 0.05; **P < 0.01; ***P < 0.001. ε1: dependent variable

SEM predicting enhanced hemodynamic fluctuations in preoperative patients is shown in Fig. 2 and Table 4. The path coefficients (r) indicated the direction and magnitude of the associations. Age (*r* = − 0.0113, 95% CI = − 0.0126–0.010, *P* < 0.001), ASA-PS score (*r* = − 0.0788, 95% CI = − 0.1431–0.0145, *P* = 0.02), sex (*r* = 0.057, 95% CI = 0.0149–0.9906, *P* = 0.01), and fentanyl dose (*r* = 0.1087, 95% CI = 0.0707–0.1467, *P* < 0.001) were significant factors that influenced the dose of propofol directly. The OR estimated using logistic regression in SEM of enhanced hemodynamic fluctuations was significantly increased per age increment (1.008, 95% CI = 1.001–1.015, *P* = 0.03). Conversely, ischemic heart disease (OR = 1.54, 95% CI = 0.99–2.38, *P* = 0.06), fentanyl dose (OR = 0.83, 95% CI = 0.69–1.01, *P* = 0.06), and propofol dose (OR = 0.81, 95% CI = 0.65–1.00, *P* = 0.05) were non-significant. Based on the data in Table 4, the prediction formula of propofol dose in induction is as follows:

$$ {\displaystyle \begin{array}{l}\begin{array}{l}\mathrm{Propofol}\ \mathrm{dose}\ (mg)=\Big[2.374-0.0113\times \mathrm{age}\ \left(\mathrm{year}\right)-0.0788\\ {}\left(\mathrm{if}\ \mathrm{ASA}- PS\ 3\  or\ 4\right)+0.057\ \left(\mathrm{if}\ \mathrm{female}\right)+0.1087\end{array}\\ {}\times \mathrm{fentanyl}\ \mathrm{dose}\ \left(\upmu \mathrm{g}/\mathrm{kg}\right)\Big]\times \mathrm{body}\ \mathrm{weight}\ \left(\mathrm{kg}\right)\end{array}} $$

**Table 4:** Structural equation model of enhanced hemodynamic fluctuation (≥30%)

Variables	Model coefficient (95% CI)	*P* value
Propofol dose	Regression coefficient (r)	
Age (year, continuous)	−0.0113 (− 0.0126–0.0100)	< 0.001
ASA-PS scale	−0.0788 (− 0.1431–0.0145)	0.02
Sex	0.0570 (0.0149–0.9906)	0.01
Ischemic heart disease	−0.0372 (− 0.1388–0.0645)	0.47
Fentanyl (μg/kg)	0.1087 (0.0707–0.1467)	< 0.001
Constant	2.374 (2.275–2.473)	
Enhanced hemodynamic fluctuation (≥30%)	OR (95% CI)	P value
Age (year, continuous)	1.008 (1.001–1.015)	0.03
Ischemic heart disease	1.536 (0.990–2.382)	0.06
Fentanyl (μg/kg)	0.831 (0.687–1.005)	0.06
Propofol (mg/kg)	0.807 (0.652–1.000)	0.05

*ASA-PS* American Society of Anesthesiologists physical status, *CI* Confidence interval, *OR* Odds ratio

As a result of the application of this formula to a validation cohort, the prevalence of hemodynamic fluctuations in the unfitted group (26.7%, *n* = 1654) was significantly higher than that in the fitted group (19.7%, *n* = 320) (*P* = 0.009). Since all VIF values were lower than 2, we confirmed the absence of multicollinearity (data not shown).

## Discussion

In our study, the prevalence of hemodynamic fluctuations after induction of general anesthesia was observed to be 23.0%. Age (OR = 1.008, 95% CI = 1.001–1.015, *P* = 0.03) was significantly associated with enhanced hemodynamic fluctuations. Whereas, ischemic heart disease (OR = 1.54, 95% CI = 0.99–2.38, *P* = 0.06), fentanyl dose (OR = 0.83, 95% CI = 0.69–1.01, *P* = 0.06), and propofol dose (OR = 0.81, 95% CI = 0.65–1.00, *P* = 0.05) were marginally associated. Conversely, age (*r* = − 0.0113, 95% CI = − 0.0126–0.010, *P* < 0.001), ASA-PS score (*r* = − 0.0788, 95% CI = − 0.1431–0.0145, *P* = 0.02), sex (*r* = 0.057, 95% CI = 0.0149–0.9906, *P* = 0.01), and fentanyl dose (*r* = 0.1087, 95% CI = 0.0707–0.1467, *P* < 0.001) were significant factors that influenced the dose of propofol directly.

A recent study found that the prevalence of hypotension (SBP < 80 for > 5 min) and hypertension (SBP > 160 for > 5 min) were 26 and 20%, respectively [26]. Similarly, 25% of patients had arterial blood pressure fluctuations greater than 30% during anesthesia induction using propofol [27]. Therefore, the prevalence in our study of hemodynamic fluctuations after induction of general anesthesia was similar to the prevalence reported in other studies.

The recommended dose of propofol in induction is 2–2.5 mg/kg. Since geriatric patients and patients with ASA-PS scores of 3 or higher are more sensitive to the anesthetic and adverse effects, a dose reduction is recommended. In fact, predictors of hypotension after induction were ASA-PS score of 3 or 4 and age older than 50 years [1]. Also, age 65 years or older and higher dose of propofol were major two highly significant predictive factors for hypotension caused by propofol injection [2]. Additionally, the favorable induction dose of propofol in patients younger than 60 years was 2.25–2.5 mg/kg, whereas in patients older than 60 years was 1.5–1.75 mg/kg [21]. The induction dose of propofol should be adjusted for an individual patient’s condition.

To determine the suitable dose of propofol to protect against hemodynamic fluctuations, we investigated the relation of propofol dose in induction to hemodynamic fluctuations. As shown in Table 4, age, sex, ASA-PS score, and fentanyl dose were associated with propofol dose to stabilize hemodynamic responses in induction.

Previous studies have provided consistent evidence for relationships between age and ASA-PS and enhanced hemodynamic fluctuation [1, 2, 21]. On the other hand, there was no difference that the effect of sex on the propofol dose in relation to loss of consciousness [28]. Similarly, a logistic regression model was used to predict blood pressure change caused by rapid injection of propofol during anesthesia induction [27]. It found that sex did not significantly contribute to blood pressure change. However, females were more likely to become hypotensive than males [2] and the half maximal effective concentration of propofol in males was significantly higher than females [29]. In our study, sex was not related to hemodynamic changes, but females required higher doses of propofol in induction than males.

Increasing the induction dose of fentanyl was a predictive factor for hypotension [1]. Similarly, concomitant use of opioids was a minor risk factor for hypotension caused by propofol [2]. Conversely, five μg/kg of fentanyl treatment caused a significant attenuation of the blood pressure response to laryngoscopy and intubation [30]. The magnitude of post-intubation hypertension was reported to be significantly less according to an increase in the fentanyl dose (*P* < 0.05 for 2 or 4 μg/kg vs. no fentanyl; *P* < 0.05 for 4 μg/kg vs. 2 μg/kg) [31].

Fentanyl was shown to cause and promote hypotension [1, 2]. On the other hand, the addition of fentanyl prior to propofol induction is used to decrease propofol dose and hypertensive response to tracheal intubation [30–32]. In our study, 1.3 μg/kg of fentanyl for the stable group (MAP change < 30%) and 1.2 μg/kg for the unstable group (MAP change ≥30%) were administered for anesthetic induction (*P* < 0.001). This result suggests that the preventive effect of fentanyl on hypertension is more dominant than the promoting effect of fentanyl on hypotension. Consequently, the propofol dose in induction increased with the increasing fentanyl dose (Table 4 and Fig. 2). Therefore, our formula indicating a positive association between propofol dose and fentanyl dose may be biologically plausible.

Our retrospective cohort study has several limitations. Generally, the researchers retrospectively identify the exposure and the outcome information. Selection bias would be possible if participation had been influenced by the physical status of patients. However, patients who had severe comorbidities could not be induced with general anesthesia using propofol. Moreover, data were entered manually during anesthesia (such as administration of medications and time of intubation). Since post-intubation blood pressure frequently reached its highest value at 0–1 min after intubation [30, 33], it may be a less reliable estimate of the exact time of induction and intubation. Because the timing of blood pressure measurement after intubation was unfixed, it may be also less reliable. Another limitation of our study is that outcome information could not be obtained using our current data system. Cooperation with other relevant diagnosis and treatment department is crucial in the future studies.

Advantages of our single-institution study are the large size of the study population, systematic consideration of potential confounders, and uniform collection of information about anesthetic practices. To estimate the generalizable prediction formula, almost all variables that affect hemodynamic fluctuations were considered; we also included a broad spectrum of patients in terms of surgical types and comorbidities. Several similar studies examining the relation of potential predictors to hemodynamic change have been reported [19, 27, 34–36]. However, since the variables to be entered in the prediction formula were either too few or too many, this was not clinically practical. Five variables, namely age, sex, ASA-PS score, fentanyl dose, and body weight, extracted from this study can be easily obtained from any patient and are essential factors for anesthesia. Therefore, our prediction formula is readily available at the time of anesthesia. A simple, generalized formula for choosing propofol dose should be required, especially for inexperienced anesthesiologists.

## Conclusions

Age, ischemic heart disease, fentanyl dose and propofol dose were marginally or significantly associated with enhanced hemodynamic fluctuations in induction. The prediction formula derived to prevent hemodynamic fluctuations will help anesthesiologists tailor the dose of propofol in the induction of general anesthesia to each patient. Although the derived prediction formula is considered to be acceptable, future studies validating whether it can predict a patient’s risk of enhanced hemodynamic fluctuations in clinical situations are necessary.

## Abbreviations

ASA: American Society of Anesthesiologists
BMI: Body mass index
BP: Blood pressure
CI: Confidence interval
CRP: C-reactive protein
IRB: Institutional Review Board
MAP: Mean arterial blood pressure
OR: Odds ratio
PS: Physical status
SBP: Systolic blood pressure
SEM: Structural equation modeling
STROBE: Strengthening the Reporting of Observational Studies in Epidemiology
VIF: Variance inflation factor
